# A subpopulation of agouti-related peptide neurons exciting corticotropin-releasing hormone axon terminals in median eminence led to hypothalamic-pituitary-adrenal axis activation in response to food restriction

**DOI:** 10.3389/fnmol.2022.990803

**Published:** 2022-09-29

**Authors:** Ruksana Yesmin, Miho Watanabe, Adya Saran Sinha, Masaru Ishibashi, Tianying Wang, Atsuo Fukuda

**Affiliations:** ^1^Department of Neurophysiology, Hamamatsu University School of Medicine, Hamamatsu, Shizuoka, Japan; ^2^Advanced Research Facilities and Services, Preeminent Medical Photonics Education and Research Center, Hamamatsu University School of Medicine, Hamamatsu, Japan

**Keywords:** CRH neuron, paraventricular nucleus, median eminence, AgRP neuron, arcuate nucleus, NKCC1, HPA axis, corticosterone

## Abstract

The excitatory action of gamma-aminobutyric-acid (GABA) in the median-eminence (ME) led to the steady-state release of corticotropin-releasing hormone (CRH) from CRH axon terminals, which modulates the hypothalamic-pituitary-adrenal (HPA) axis. However, in ME, the source of excitatory GABAergic input is unknown. We examined agouti-related peptide (AgRP) expressing neurons in the arcuate nucleus as a possible source for excitatory GABAergic input. Here, we show that a subpopulation of activated AgRP neurons directly project to the CRH axon terminals in ME elevates serum corticosterone levels in 60% food-restricted mice. This increase in serum corticosterone is not dependent on activation of CRH neuronal soma in the paraventricular nucleus. Furthermore, conditional deletion of Na^+^-K^+^-2Cl^–^ cotransporter-1 (NKCC1), which promotes depolarizing GABA action, from the CRH axon terminals results in significantly lower corticosterone levels in response to food restriction. These findings highlight the important role of a subset of AgRP neurons in HPA axis modulation via NKCC1-dependent GABAergic excitation in ME.

## Introduction

The hypothalamic-pituitary-adrenal (HPA) axis consists of a cascade of endocrine pathways that maintain body homeostasis, including stress response modulation (Sheng et al., 2020). Corticotropin-releasing hormone (CRH) is a peptide hormone that regulates the HPA axis under both basal and stress-activated conditions. CRH is synthesized in the cell bodies of CRH neurons in the paraventricular nucleus (PVN) of the hypothalamus. Upon exposure to stress, CRH released from the axon terminals of CRH neurons in the median eminence (ME) (Palkovits, 1984), stimulates the anterior pituitary to release adrenocorticotropic hormone which activates the adrenal cortex to upregulate production of glucocorticoids (cortisol in human and corticosterone in mice) (Oakley and Cidlowski, 2013). Elevated glucocorticoid levels are regulated by a feedback mechanism (Vandenborne et al., 2005; Myers et al., 2012).

Multiple neurotransmitter systems, including noradrenergic, glutamatergic, and gamma-aminobutyric acid (GABA)-ergic synaptic inputs, regulate CRH neuronal activity. In the mature brain without stress, CRH neuronal soma receive robust GABAergic inhibition from the peri-PVN, anterior hypothalamic area, dorsomedial hypothalamic nucleus, medial preoptic area (mPOA), lateral hypothalamic area (LHA), and multiple nuclei within the bed nucleus of the stria terminalis (BNST). (Roland and Sawchenko, 1993; Tasker and Dudek, 1993; Bowers et al., 1998; Tasker et al., 1998; Miklos and Kovacs, 2002; Herman et al., 2004; Radley et al., 2009; Mody and Maguire, 2011; Levy and Tasker, 2012). However, during acute and chronic stress, GABA turns excitatory and increases CRH release (Hewitt et al., 2009; Sarkar et al., 2011; MacKenzie and Maguire, 2015; Kim et al., 2019). Inhibitory and excitatory effects of GABA are dependent on the electrochemical gradient of Cl^–^, while inhibitory GABA function is dynamically mediated by a low intracellular Cl^–^ concentration, which is regulated by K^+^-Cl^–^ co-transporter (KCC2) predominantly expressed in the cell body (Rivera et al., 1999). Conversely, under non-stressful condition in mature brain, we previously reported that excitatory GABAergic inputs originating from the arcuate nucleus (ARC) maintain steady-state CRH release from axon terminals of CRH neurons in the ME. While, excitatory GABA functions have been mediated by high intracellular Cl^–^ concentration in CRH axon terminals due to an abundance of Na^+^-K^+^-2Cl^–^ cotransporter 1 (NKCC1) rather than KCC2 (Kakizawa et al., 2016). As a result of the differential intracellular Cl^–^ levels, GABA exerts diverse physiological roles on the somata and axon terminals of CRH neurons. However, the neuronal population in the ARC that sends excitatory GABAergic inputs to CRH neuron axon terminals in the ME is still unknown.

In ARC, agouti-related peptide (AgRP)-expressing neurons produce GABA (Tong et al., 2008; Wu et al., 2009) and send their axons to the median eminence. Diverse metabolic stimuli such as fasting and peripheral orexigenic signals like ghrelin (Cowley et al., 2003; Kohno et al., 2003) activate AgRP neurons. Besides stress, fasting or caloric restriction (CR) can increase serum cortisol or corticosterone (Champy et al., 2004; Jensen et al., 2013) implying that AgRP neuronal activity and corticosterone release may have a synergistic association. However, CR did not increase the activity of the CRH neuronal soma (Kenny et al., 2014) raising the possibility that CR activated the CRH axon terminal instead of the soma, resulting in an increase in corticosterone level, as we previously demonstrated. In line with our earlier findings, here we examine AgRP neurons as a putative GABAergic source that might activates the CRH axon terminals and upregulate the HPA axis.

Thus, the aforementioned AgRP neurons projecting to the ME may play a role in HPA axis activation in response to energy depletion. Here, we set out to characterize the neuronal population that specifically projects to the ME and affects corticosterone levels in response to 60% food restriction (60% amount of food was given; 60% FR). We examined physiological roles of AgRP neuronal activation by food restriction (FR) and observed increases in circulating corticosterone levels. Thereafter, we employed a retrograde tracer to identify a subset of AgRP neurons that directly project to the ME, respond to energy homeostasis, and modify HPA axis activity to increase serum corticosterone levels via excitatory GABA action at CRH nerve terminals in the ME.

## Materials and methods

### Animals

Adult male mice (8–12 weeks old) were used to avoid sex base difference in stress response. Mice were kept in groups of 3–4 per cage under a 12-h light-dark cycle (lights off from 19:00 to 07:00) with free access to water and food pellets. AgRP-Ires-Cre mice (Stock No. 012899), CRH-Ires-Cre mice (Stock No. 012704), Cre-dependent GCaMP3 reporter mice (Ai38, Stock No. 014538), and Cre-dependent Gq-DREADD mice (CAG-LSL-Gq-DREADD, Stock No. 026220) were purchased from the Jackson Laboratory and C57BL/6J (wild-type) mice was also used. NKCC1^flox/flox^ mice (a gift from Prof. Christian A. Hübner) were previously described (Antoine et al., 2013). To visualize AgRP neurons, we crossed AgRP-Ires-Cre mice with Ai38 reporter mice to generate AgRP-Cre:GCaMP3 mice. For chemogenetic activation of AgRP neurons, we crossed AgRP-Ires-Cre mice with CAG-LSL-Gq-DREADD mice to generate AgRP Cre:DREADD mice. To generate CRH Cre:NKCC1^flox/flox^ (NKCC1 KO^CRH^) mice, we crossed CRH-Ires-Cre (NKCC1 WT) mice with NKCC1^flox/flox^ mice. Animal identifiers and mouse genotype sequences were listed in Supplementary Table 2. All experiments were performed in accordance with guidelines issued by the Hamamatsu University School of Medicine on the ethical use of animals for experimentation, and were approved by the Committee for Animal Care and Use (Approval Nos. 2017056, 2017057, 2018025, and 2020074). All efforts were made to minimize the number of animals used and their suffering.

### Food restriction

All mice were individually housed and allowed to acclimatize for 7 days before experiments. To measure the average daily food consumption, daily food intake (*ad libitum*) and body weight of WT mice was monitored for 10 days. For FR experiments, mice were given a food pellet equal to 60% of average daily food intake (60% FR). Mice were assigned to *ad libitum* (Ad-lib) or 60% FR groups for 10 days and food was added to each cage in the afternoon prior to lights off. Water was freely available to both groups. To minimize the circadian variation in feeding and corticosterone levels, mice for all experiments were sacrificed between 09:00 and 11:00 a.m. Trunk blood was collected for hormone analysis, while brains were collected for c-Fos immunostaining.

### Retrograde labeling

To label AgRP neurons sending axon terminals to the ME, AgRP-Cre:GCaMP3 mice received an intraperitoneal (i.p.) injection of the retrogradely transportable marker compound Fluoro-Gold (40 mg/kg; Fluorochrome). Because ME is the circumventricular organ, the uptake of the retrograde tracer and its accumulation in cell bodies occurs following injection into the bloodstream (Swanson and Kuypers, 1980). Four days after injection, mice were transcardially perfused with phosphate-buffered saline (PBS), followed by a 4% paraformaldehyde solution (PFA), and then brains were collected for immunohistochemistry.

### Chemogenetic stimulation

For chemogenetic activation of AgRP neurons, AgRP-Cre:DREADD mice were treated with clozapine N-oxide (CNO; 1 mg/kg B.W.). For acute studies, food intake and body weight were measured at 0, 1, 2, and 24 h after saline/CNO injection. For the hormone assay, blood samples were collected 1 h after CNO or 0.9% saline i.p. injection between 09:00 and 11:00 a.m. For c-Fos immunohistochemistry, brain tissue was collected 90 min after CNO or 0.9% saline injection.

### Immunohistochemistry

Mice were deeply anesthetized with sodium pentobarbital (50 mg/kg) and transcardially perfused with PBS, followed by ice-cold 4% PFA. Brains were collected and placed in 4% PFA for 2 h, followed by 20 and 30% sucrose in 0.1 M phosphate buffer (PB) at 4°C. Next, brains were frozen, coronally sectioned (30 μm), and processed for immunohistochemistry as previously described (Kakizawa et al., 2016). Free-floating sections were washed in 0.1% Tween 20 in PBS (PBS-T), and then blocked using 10% normal goat serum or normal donkey serum in PBS-T at room temperature. Subsequently, sections were incubated for 24–48 h with the following primary antibodies diluted in PBS-T at 4°C: guinea pig anti-CRH (1:800; Peninsula Laboratories, Carlos, CA, USA, T-5007), mouse anti-c-Fos (1:2000; Abcam, ab208942), chicken anti-GFP (1:1000; Abcam, ab13970), goat anti-AgRP (1:1000; R&D system, AF634), rabbit anti-TH (1:3000; Millipore, Ab152), mouse anti-HA (1:1000; Biolegend, 901501), rabbit anti-c-Fos (1:3000; Sigma-Aldrich, F7799), mouse monoclonal anti-NKCC1 (T4) (1:3000, DSHB), rabbit anti-POMC (1:2000; Phoenix Pharmaceuticals, Inc., Burlingame, CA, USA H-029-30), rabbit anti-Fluoro-Gold (1:500; Merck, AB 153-I). Antibody unique Identifiers and their resources were listed in Supplementary Table 2. Subsequently, all floating sections were washed several times with PBS-T and incubated with the following secondary antibodies: Alexa Fluor 488-conjugated goat anti-guinea pig IgG, Alexa Fluor 594-conjugated goat anti-guinea pig IgG, Alexa Fluor 594-conjugated goat anti-mouse IgG, Alexa Fluor 647-conjugated goat anti-mouse IgG, Alexa Fluor 488-conjugated goat anti-chicken IgG, Alexa Fluor 488-conjugated donkey anti-goat IgG, Alexa Fluor 488-conjugated goat anti-rabbit IgG, and Alexa Fluor 594-conjugated goat anti-rabbit IgG, (1:1000, all from Molecular Probes) at room temperature for 2 h. After several washes with PBS-T, sections were mounted and cover slipped. Confocal microscopy (Olympus FV1000-D or Leica TCS SP8) was used to capture images.

### Cell counting

To determine whether FR induces c-Fos in AgRP neurons, AgRP-Cre:GCaMP3 mice were evaluated and CNO-mediated c-Fos induction was examined in AgRP-Cre:DREADD mice. For cell quantification, ImageJ (Fiji, NIH) was used to define a specific region of interest, such as the PVN or ARC. Four to five representative sections from one or both sides of the PVN and ARC of each mouse were included in this study. To distinguish cells from background, thresholding was adjusted to minimize non-specific background fluorescence. Individual neurons expressing AgRP or CRH were identified and counted by adjusting threshold values. c-Fos-positive cells were also counted in 30-μm coronal sections obtained serially across the anterior to posterior axis of the PVN and ARC of the hypothalamus using the “analyze particles” feature, such that consistent fluorescence and size thresholds were used throughout, as previously described (Grishagin, 2015).

### Electron microscopic analysis

Mice were anesthetized with sodium pentobarbital (50 mg/kg) and perfused with 0.1 M PB containing 4% PFA and 0.5% glutaraldehyde. Following perfusion, the brain tissue was quickly cut into 1-mm square pieces with a razor and fixed with same solution for 2 h at 4°C. Next, the brain tissue was rinsed three times (10 min each) in 0.1 M PB to completely remove the fixative agents and post-fixed in 0.1 M PB containing 1% osmium tetroxide for 2 h at room temperature. Next, the fixed brain tissue was dehydrated by incubation in a series of ethanol solutions, followed by propylene oxide. Following dehydration, specimens were placed in a mold filled with liquid resin and cured into a hard block using heat. Samples embedded in London Resin (LR) white acrylic resin were subsequently polymerized at 48°C for 1 day and 60°C for 2 days. Each polymerized block was cut into semithin sections (1 μm) with a glass knife using an ultramicrotome. After staining the semithin sections with toluidine blue, trimming was performed and ultrathin (80-nm) sections made using a diamond knife were collected on nickel grids. Next, the ultrathin sections were subjected to double immunostaining for CRH and AgRP using gold-conjugated secondary antibodies. Primary antibodies and gold conjugates were diluted in Tris-buffered saline (TBS, pH 7.4) containing 1% bovine serum albumin (BSA) and 0.1% Tween 20. Grids with ultrathin section were placed on a drop of blocking solution in TBS containing 1% BSA, 0.1 M glycine, and 0.1% Tween 20 for 1 h at room temperature. Next, grids with sections were incubated overnight at 4°C with guinea pig anti-CRH (1:100; Peninsula Laboratories, Carlos, CA, USA, T-5007) and goat anti-AgRP (1:100; R&D Systems, AF634) antibodies. All grids were then washed with a few drops of TBS and incubated with 6-nm colloidal gold-donkey anti-goat IgG and 18-nm colloidal gold-donkey anti-guinea pig IgG secondary antibodies (Jackson Immuno Research Laboratories, West Grove, PA, USA) for 1 h at room temperature. Next, grids were stained with uranyl acetate for 5 min and lead citrate for 3 min. Observations were made with a transmission electron microscope (JEM 1400, JEOL) and images were recorded on a charge-coupled device camera.

### Hormone assay

Adult mice were sacrificed by cervical dislocation. Mice were decapitated and the trunk blood was collected into polyethylene tubes containing EDTA-2K (Becton, Dickinson and Company, Franklin Lakes, NJ, USA) and centrifuged at 3000 rpm for 20 min at 4°C. Subsequently, serum was collected and stored at −80°C until use in the hormone assay. Samples were collected between 09:00 and 11:00 a.m. to minimize the effect of circadian rhythm. Plasma corticosterone levels were determined using a radioimmunoassay, as previously reported (Uchida et al., 2011). Briefly, a 25-μl sample of serum was boiled at 98°C for 5 min. Ice-cold radioimmunoassay buffer [0.1 M PB (pH 7.4) containing 0.05% NaN_3_ and 0.1% Triton X-100] was used for dilution. ^125^I-corticosterone (Institute of Isotopes) was used as the label. A mixture comprising 100 μl of corticosterone standard or sample, 100 μl of corticosterone antiserum, and 100 μl of ^125^I-labeled corticosterone was incubated for 24 h at 4°C. Antibody-bound and antibody-free corticosterone were separated via incubation with 100 μl of a secondary antibody (bovine γ-globulin) and 400 μl of 25% polyethylene glycol, followed by centrifugation at 3000 rpm for 15 min at 4°C. Radioactivity of label bound to the antibody was counted using a γ-counter (ARC-7010, Aloka, Tokyo, Japan). The assay did not cross-react with other corticosteroids and the sensitivity was 2 pg/tube.

### Statistics

Statistical analysis was performed using GraphPad Prism software. Data were first assessed for normality of distribution by the Kolmogorov-Smirnov test. For comparison of two groups, unpaired Student’s *t* test was used. ANOVA (one- or two-way) followed by Tukey’s or Bonferroni *post hoc* tests were used to analyze multiple comparisons. Statistical significance was defined as *P* < 0.05. All data are presented as the mean ± SEM.

## Results

### Agouti-related peptide and corticotropin-releasing hormone neuronal terminal juxtaposition in median-eminence

**FIGURE 1:**
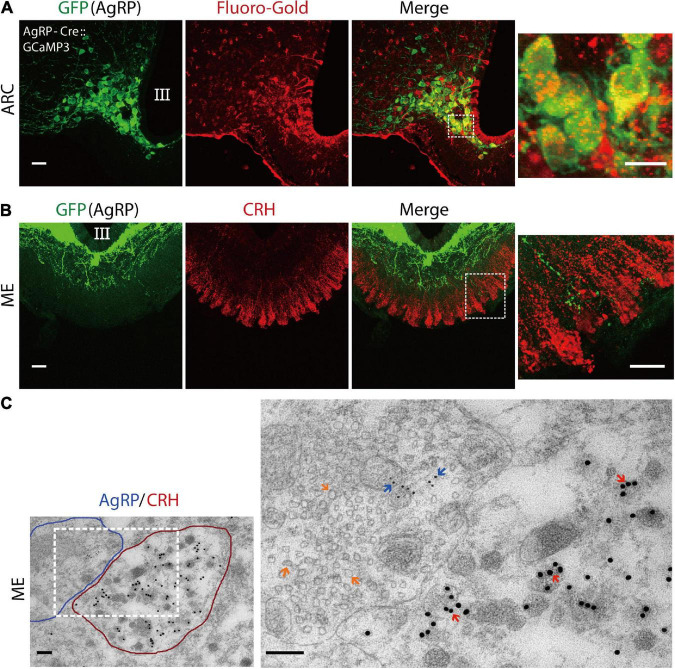
Agouti-related peptide (AgRP) neurons project to corticotropin-releasing hormone (CRH) neuronal terminals in the median eminence. **(A)** The retrograde tracer Fluoro-Gold was peripherally delivered in AgRP-Cre:GCaMP3 mice. Confocal image showing AgRP neurons in the arcuate nucleus (ARC) stained with an anti-GFP antibody (for GCaMP3; green) and anti-Fluoro-Gold antibody (red). The dotted boxed area indicates higher magnifications image. Scale bars, 40 and 10 μm (box). **(B)** Confocal image showing anti-GFP (green) and anti-CRH (red) immunoreactivity in the median-eminence (ME). Higher magnification image indicated by box shows AgRP and CRH neuronal axon terminals in the external layer of the ME. Scale bars, 40 and 10 μm (box). **(C)** Double immuno-electron microscopic images showing an AgRP nerve terminal (outlined in blue) contacting a CRH neuronal terminal (outlined in red) in the ME of wild-type mice. Note that the AgRP neuronal axon terminal makes direct contact to the axon terminal of the CRH neuron. Inset box image shows higher magnification image. AgRP immunoreactivity (6-nm gold particle) indicated by blue arrow and CRH immunoreactivity (18-nm gold particle) indicated by red arrow in the external layer of the ME. Large dense-core vesicles indicate CRH-containing vesicles, while small clear vesicles marked by orange arrows in AgRP neuronal terminal indicate gamma-aminobutyric-acid (GABA)-containing vesicles. Scale bars, 500 and 200 nm, respectively. III, third ventricle.

Emerging evidence suggests that AgRP neurons located in the ARC of the hypothalamus are GABAergic (Tong et al., 2008; Wu et al., 2009). Previously, we reported that GABAergic input originates from the ARC in the ME where the terminals of CRH neurons exist (Kakizawa et al., 2016). So, we postulated that if AgRP contributes to excitatory GABAergic input, it must project from the ARC to the ME, where it will be juxtaposed with the CRH axon terminal. Therefore, we examined the projection of AgRP neurons to ME. To visualize AgRP neurons in the ARC, we crossed AgRP-Cre mice with GCaMP3 (Ai38) reporter mice to generate AgRP-Cre:GCaMP3 mice expressing GCaMP3 with enhanced green fluorescent protein (EGFP) in Cre-expressing AgRP neurons. Fluoro-Gold, a retrograde tracer that does not penetrate the blood-brain barrier, was administered peripherally. Injection (i.p.) of Fluoro-Gold retrogradely labeled AgRP neurons in the ARC of AgRP-Cre:GCaMP3 mice (*n* = 3 mice; Figure 1A). Next, we performed double immunostaining for EGFP (AgRP) and CRH. The results show that AgRP neurons densely projected over the internal layer of the ME with some projections extending to the external layer, whereby AgRP axon terminals were juxtaposed to CRH axon terminals (*n* = 4 mice; Figure 1B). For further confirmation, we performed immuno-electron microscopy. As shown in Figure 1C, the CRH neuron terminal (encircled with red) was juxtaposed to an AgRP neuron terminal (encircled with blue) in the external layer of ME. CRH immunoreactivity (large 18-nm gold particles) was confined to dense-core secretory granules, which have a mean diameter of approximately 90–100 nm. AgRP neuron terminals contained both large dense-core vesicles containing AgRP immunoreactivity (small 6-nm gold particles) and small clear vesicles, indicative of GABA vesicles. The higher magnification image shows that the AgRP neuron axon terminal had symmetric synaptic contact with the CRH neuronal terminal (*n* = 3 mice; Figure 1C). Together, these data suggest that GABA-containing AgRP neuronal terminals contact CRH axon terminals at the external layer of the ME.

### Chemogenetic activation of agouti-related peptide neurons resulted in the increase in serum corticosterone levels

To test our hypothesis that AgRP neuronal activation contribute to increased serum corticosterone levels, we employed an excitatory hM3Dq designer receptor exclusively activated by designer drugs (DREADD)-mediated chemogenetic approach to specifically activate AgRP neurons. AgRP-Ires-Cre mice were crossed with hM3Dq DREADD mCitrine mice to generate AgRP-Cre:DREADD transgenic mice (Supplementary Figure 1A). To verify specific expression of hM3Dq DREADD in AgRP neurons after Cre recombination, brain sections of AgRP Cre:DREADD mice were immunohistochemically evaluated for expression of the yellow-green fluorescent protein mCitrine and hemagglutinin (HA) epitope tag. GFP antibody was used to stain mCitrine reporter (*n* = 4 mice for AgRP/GFP and *n* = 4 mice for HA/GFP; Supplementary Figure 1B).

**FIGURE 2:**
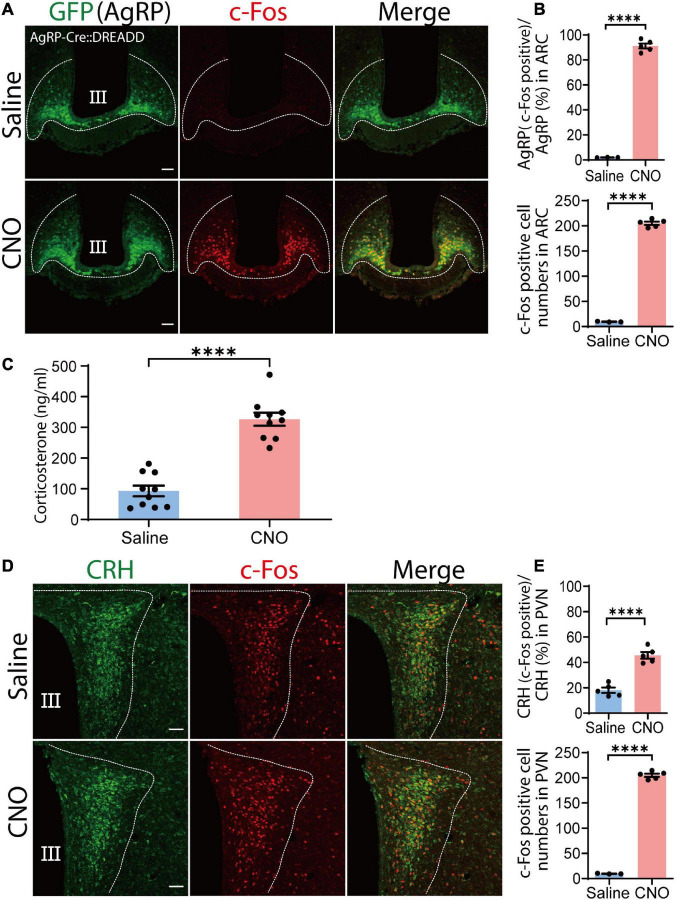
Chemogenetic activation of agouti-related peptide (AgRP) neurons by clozapine N-oxide (CNO) causes corticotropin-releasing hormone (CRH) neuronal somatic activation and increased serum corticosterone levels. **(A)** Injection of CNO i.p. induced c-Fos expression in the arcuate nucleus (ARC). Confocal image showing AgRP neurons stained with an anti-GFP antibody (green) and anti-c-Fos antibody (red). Scale bars, 50 μm. **(B)** Percentage of AgRP neurons expressing c-Fos in the ARC in upper panel; and total numbers of c-Fos-positive cells in the ARC in lower pannel; ****P < 0.0001, unpaired Student’s *t* test. **(C)** Serum corticosterone level 1 h after CNO and saline injection; ****P < 0.0001, unpaired Student’s *t* test. **(D)** Confocal image showing c-Fos expression in CRH neurons stained with an anti-CRH antibody (green) in the paraventricular nucleus (PVN) area after CNO and saline injection. Scale bars, 50 μm. **(E)** Proportion of CRH and c-Fos colocalization in the PVN **(upper panel)**. In total, 86.76 ± 3 CRH cells colocalized with c-Fos out of a total of 192 ± 3 CRH cells; ****P < 0.0001, unpaired Student’s *t* test. Quantification of total c-Fos expression in the PVN; ****P < 0.0001, unpaired Student’s *t* test **(lower panel)**. Error bars represent SEM. Dotted line indicate the area for counting of AgRP, CRH and c-Fos positive cells.

Function of the excitatory DREADD was verified by specific expression of DREADD ligand clozapine N-oxide (CNO)-induced c-Fos expression in AgRP neurons. Following CNO administration, 91.1% ± 2% of AgRP neurons expressed c-Fos (195.08 ± 4.95 c-Fos-positive AgRP cells/214.04 ± 4.95 total AgRP cells), indicating good efficacy of our DREADD-mediated approach (saline, *n* = 3 mice, four sections per mouse; CNO, *n* = 5 mice, five sections per mouse; *P* < 0.0001, unpaired Student’s *t* test; Figures 2A,B). We also observed that food intake was significantly increased by CNO administration without immediately significant body weight change (Supplementary Figures 2A,B). These results indicate that AgRP neurons were sufficiently activated. Next, we tested the potential involvement of AgRP neurons in increased levels of circulating corticosterone following i.p. injection of CNO or saline. We observed that CNO effectively increased serum corticosterone levels compared with the saline-treated group (saline, 92.84 ± 17.30 ng/ml; CNO, 326.5 ± 21.13 ng/ml; *n* = 10 mice per group; *P* < 0.0001, unpaired Student’s *t* test; Figure 2C). These results demonstrate that the observed increase of serum corticosterone induced by CNO was dependent on ARC AgRP neuron activation.

Generally, increases in serum corticosterone levels are dependent on CRH neuronal activation of the PVN (Kim et al., 2019). Thus, we next examined c-Fos expression of CRH neurons in the PVN by chemogenetic activation of AgRP neurons. CNO administration significantly increased c-Fos expression in CRH neurons compared with saline administration, indicating that CRH neuronal activation caused corticosterone secretion. Indeed, we found that 45.58% ± 1.86% of CRH neurons in the PVN area were positive for c-Fos in the CNO-treated group, significantly more than observed in the saline-treated group 18.08% ± 1.20% (*P* < 0.0001; unpaired Student’s *t* test; *n* = 5 mice, five sections per mouse; Figures 2D,E). This result indicates that chemogenetic activation of AgRP neurons activates CRH neuronal somata in the PVN.

### Physiological activation of agouti-related peptide neurons activates the corticotropin-releasing hormone axon terminals, resulting in increased blood corticosterone levels

**FIGURE 3:**
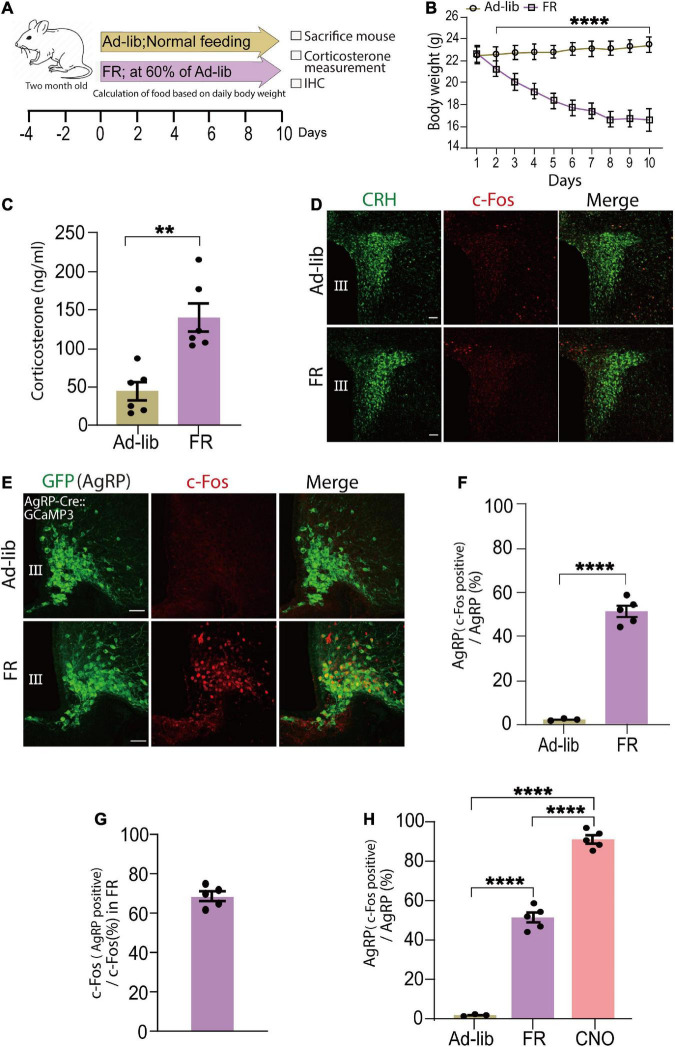
Food restriction (FR) activates agouti-related peptide (AgRP) neurons and increases serum corticosterone levels. **(A)** Schematic diagram of 60% FR for 10 days. FR was applied every day at 6 p.m. in wild-type adult male mice. **(B)** FR significantly decreased body weight; ****P < 0.0001, unpaired Student’s *t* test. **(C)** Plasma corticosterone levels at 9.00 a.m.–11.00 a.m. was significantly increased in the FR group; **P < 0.0013; unpaired Student’s *t* test. **(D)** c-Fos expression was not observed in the PVN area of *ad libitum* (Ad-Lib) or FR groups. Scale bars, 50 μm. **(E)** Confocal image showing c-Fos expression in AgRP neurons in the arcuate nucleus (ARC) stained with an anti-GFP antibody in AgRP-Cre:GCaMP3 mice. Scale bars, 40 μm. **(F)** Expression of c-Fos in AgRP neurons (51.3% ± 1.7%) was significantly increased by FR; ****P < 0.0001, unpaired Student’s *t* test. **(G)** Approximately 68.53% ± 2.04% of c-Fos-expressing neurons were AgRP neurons in ARC of FR group. **(H)** AgRP neurons expressing c-Fos in the ARC after clozapine N-oxide (CNO) injection (91.1% ± 2%) were significantly more than that by FR; ****P < 0.0001; unpaired Student’s *t* test. Error bars represent SEM.

As AgRP neurons are progressively activated by energy deficient signal or FR (Betley et al., 2015; Chen et al., 2015), next as physiological stimulus we examined whether FR can increase serum corticosterone levels. In this study, we defined FR group mice as those who were only given 60% of the food compared to control (Ad-lib) mice (Figure 3A). As expected, when compared to the Ad-lib group, 60% of FR for 10 days resulted in a significant weight loss that was visible as early as the 2 day (*n* = 6 mice per group, *P* < 0.005; Figure 3B). Next, we investigated whether 60% FR had any effect on serum corticosterone levels via AgRP cell activation in adult wild-type mice. Consistent with a previous report (Allen et al., 2019) serum corticosterone levels were significantly increased following 60% FR compared with level of the Ad-lib group (Ad-lib, 43.93 ± 11.5 ng/ml; FR, 140.4 ± 18.6 ng/ml, *n* = 6 mice per group; *P* < 0.0013, unpaired Student’s *t* test; Figure 3C). Increased serum corticosterone levels are generally dependent on CRH neuronal activation in the PVN (Parton et al., 2007). Accordingly, we examined c-Fos expression in CRH neuronal somata in the PVN. Interestingly, in contrast to chemogenetic activation of AgRP neurons, no c-Fos expression was observed in the PVN area (Figure 3D), indicating that increased serum corticosterone by 60% FR does not result from somatic activation of CRH neurons in the PVN.

Fasting is a catabolic-metabolic state that causes several physiological changes in mice, such as activation of AgRP neurons in the ARC (Takahashi and Cone, 2005; Liu et al., 2012). CR upregulates AgRP mRNA expression level (Rogers et al., 2016). Thus, next we examined c-Fos expression in AgRP neurons to detect cells activated by FR. Following 60% FR in AgRP-Cre:GCaMP3 mice to clearly visualize AgRP neurons, c-Fos expression was significantly increased (Figure 3E). Specifically, we observed that 51.3% ± 1.7% of AgRP neurons (108.6 ± 4.73 c-Fos positive AgRP cells/211.76 ± 5.86 total AgRP cells) were activated (c-Fos positive; Figure 3F) and 68.53% ± 2.04% of c-Fos-positive cells were AgRP neurons under 60% FR (Figure 3G) (*n* = 5 mice, five sections per mouse; *P* < 0.0001, unpaired Student’s *t* test).

We next evaluated proportions of activated AgRP neurons following CNO administration and FR. CNO treatment activated significantly more AgRP neurons (91.1% ± 2%) compared with 60% FR (51.3% ± 1.7%; *P* < 0.0001; unpaired Student’s *t* test; *n* = 5 mice, five sections per mouse; Figure 3H). Furthermore, numbers of c-Fos-expressing neurons (CNO, 205.12 ± 5; FR, 159.6 ± 6.11; Supplementary Figure 3B), c-Fos-expressing AgRP neurons (CNO, 195.08 ± 4.95; FR, 108.6 ± 4.73; Supplementary Figure 3C) in the ARC and serum corticosterone levels (CNO, 326.50 ± 21.13 ng/ml; FR, 140.4 ± 18.64 ng/ml; Supplementary Figure 3D) were significantly higher in the CNO-treated group compared with the 60% FR group. Collectively, these data indicate that about half of ARC AgRP neurons responded to FR and played an active role in modulation of circulating corticosterone levels. Because of the observed lack of activation of PVN CRH neurons by 60% FR, we hypothesize this subgroup may be responsible for modulating CRH release at the ME.

### A subset of activated agouti-related peptide neurons directly project to the median eminence

**FIGURE 4:**
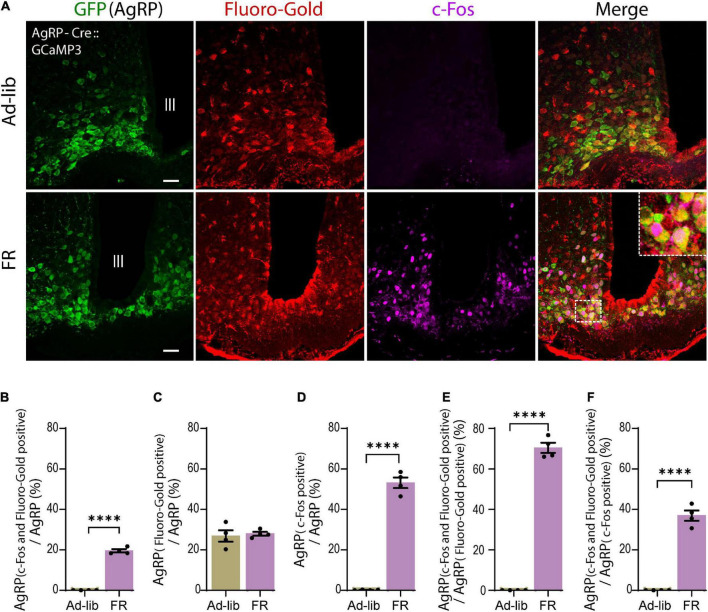
A subset of agouti-related peptide (AgRP) neurons directly projecting to the median eminence were activated by food restriction (FR). **(A)** Triple immunostaining of arcuate nucleus (ARC) with anti-GFP for AgRP (green), anti-Fluoro-Gold (red), and anti-c-Fos (magenta) antibodies in *ad libitum* (Ad-lib) **(upper panel)** and FR **(lower panel)** conditions. Note that no c-Fos signals were observed in Ad-lib group. The dotted boxed area indicates higher magnifications image (× 4). Scale bars, 40 μm. **(B)** A subset of AgRP neurons (19.49% ± 0.82%) co-express both Fluoro-Gold and c-Fos under FR. **(C)** In the FR, 27.72% ± 0.95% of AgRP neurons were labeled by Fluoro-Gold, while comparable proportion of AgRP neurons (26.76% ± 2.73%) were labeled by Fluoro-Gold in Ad-lib group (*P* = 0.737; unpaired Student’s *t* test). **(D)** 53.43% ± 3% AgRP neurons were c-Fos positive in FR group. **(E)** 70.38% ± 2.54% of Fluro-Gold-positive AgRP neurons were also positive for c-Fos in FR group. **(F)** 36.84% ± 2.58% of c-Fos-positive AgRP neurons were Fluoro-Gold positive. ****P < 0.0001; unpaired Student’s *t* test. Error bars represent SEM.

Agouti-related peptide neurons can be subdivided based on their projection pattern (Betley et al., 2013). Thus, we examined the population of AgRP neurons that projected directly to the ME. To reveal this, triple-immunostaining for AgRP, Fluoro-Gold, and c-Fos was performed (Figure 4A). We found that 19.49% ± 0.82% of AgRP neurons were double positive for Fluoro-Gold and c-Fos under 60% FR (Figure 4B). We also observed that 27.72% ± 0.95% of AgRP neurons were labeled by Fluoro-Gold in FR group (Figure 4C) as comparable to the control Ad-lib group, indicating that this population of AgRP neurons may directly project to the ME. We also observed that 53.43% ± 3% of AgRP neurons expressed c-Fos by 60% FR (Figure 4D). Thus, we found that 70.38% ± 2.54% of Fluoro-Gold-positive AgRP neurons were also positive for c-Fos after 60% FR (Figure 4E). In other words, most AgRP neurons directly projecting to the ME were activated by FR. Furthermore, 36.84% ± 2.58% of c-Fos-positive AgRP neurons were labeled by Fluoro-Gold (Figure 4F), indicating more than one third of FR-responding AgRP neurons are ME-projecting ones (*n* = 4 mice, five sections of one side of the ARC per mouse in each group from Figures 4A–F). Numbers of cells expressing: AgRP, c-Fos and AgRP, c-Fos, Fluoro-Gold and AgRP in FR group are shown in Supplementary Table 1.

It has been established that several types of GABAergic neurons in the ARC regulate energy homeostasis (Cone et al., 2001). Other studies show that tyrosine hydroxylase (TH)-positive ARC neurons are GABAergic and activated by overnight fasting (Zhang and van den Pol, 2015; Zhang and van den Pol, 2016). Following 60% FR, TH neurons exhibited almost no c-Fos expression (Supplementary Figure 4A). Proopiomelanocortin (POMC) neurons are the main anorexigenic neuronal population, thus we next examined c-Fos expression in POMC neurons (Supplementary Figure 4B). As expected, POMC neurons did not exhibit c-Fos expression after FR. Alternatively, our data suggest that 60% FR activated 31.47% ± 2.37% of non-AgRP neurons in the ARC, excluding TH and POMC neurons. However, these groups of c-Fos-positive non-AgRP neurons were not labeled by Fluoro-Gold (see Figure 4A; *n* = 4 mice, five sections per mouse) indicating no projection to the ME. All together, these data indicate that the majority of AgRP neurons directly projecting to the ME are activated under FR, whereby they may mediate FR-induced modulation of CRH release at ME.

### Conditional deletion of NKCC1 from corticotropin-releasing hormone neuron terminals reduced serum corticosterone elevation induced by food restriction

**FIGURE 5:**
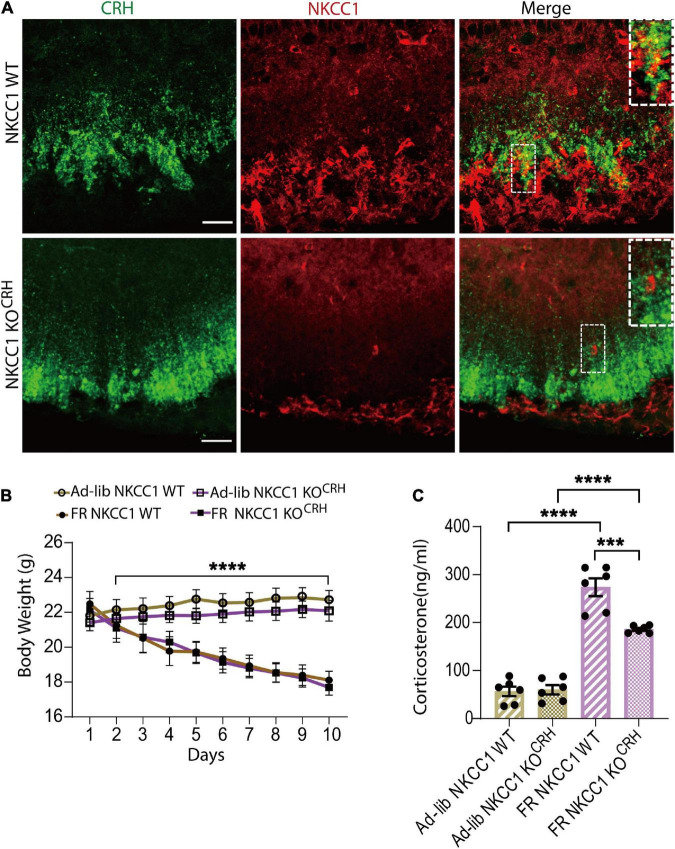
Conditional deletion of NKCC1 from corticotropin-releasing hormone (CRH) neuronal terminals reduced the elevation of serum corticosterone in response to food restriction. **(A)** Immunohistochemical staining with anti-CRH (green) and anti-NKCC1 (red) antibodies in CRH neuronal terminals in the median-eminence (ME). NKCC1 immunoreactivity was observed in the outer layer of the ME and colocalized with CRH (upper panel). In NKCC1 KO^CRH^ mice, NKCC1 immunoreactivity was not observed in CRH neuronal terminals (lower panel). Square areas at upper right corner are enlarged image of dotted area. Scale bars, 40 μm. **(B)** Temporal changes in body weight in *ad libitum* (Ad-lib) and food restriction (FR) conditions; ****P < 0.0001; two-way ANOVA with Tukey’s multiple comparison test. **(C)** Concentrations of serum corticosterone at 9:00–11:00 a.m. in Ad-lib and FR groups, indicating a significant reduction in FR-induced corticosterone increases in NKCC1 KO^CRH^ compared with NKCC1 WT; ****P < 0.0001, ***P < 0.0003, two-way ANOVA with Bonferroni’s multiple comparison test. Error bars represents SEM.

Gamma-aminobutyric-acid-mediated depolarization is dependent on the high intracellular Cl^–^ concentration produced by NKCC1 (Yamada et al., 2004). CRH neuronal axon terminals, but not soma, express NKCC1. GABA evokes an increase of intracellular Ca^2+^ in CRH neuron terminals that is attenuated by the NKCC1 inhibitor bumetanide (Kakizawa et al., 2016). To establish whether NKCC1 is involved in CRH release from axon terminals of CRH neurons to cause FR-induced corticosterone increases, we generated conditional NKCC1-knockout mice by crossing CRH-Cre mice with NKCC1^flox/flox^ mice. Using the resulting CRH-Cre:NKCC1^flox/flox^ (NKCC1 KO^CRH^) mice, we confirmed deletion of NKCC1 from CRH axon terminals by immunohistochemical analysis (*n* = 3 mice; Figure 5A). Body weights were significantly decreased in both control (NKCC1 WT) and conditional NKCC1 KO^CRH^ groups following 60% FR (*n* = 6 mice per group; Figure 5B). Next, we measured serum corticosterone levels. Under *ad libitum* conditions, there were no differences in serum corticosterone levels between control and conditional NKCC1 KO^CRH^ groups (56.48 ± 10.13 ng/ml and 59.73 ± 9.67 ng/ml, respectively; *n* = 6 mice per group; *P* > 0.999; two-way ANOVA with Bonferroni’s multiple comparisons test; Figure 5C). However, serum corticosterone levels in NKCC1 KO^CRH^ mice were significantly reduced compared with control mice following FR (186.03 ± 3.07 ng/ml and 273.8 ± 18.59 ng/ml, respectively; *n* = 6 mice per group; *P* < 0.0003, two-way ANOVA, Bonferroni’s multiple comparisons test; Figure 5C). Together, these data indicate that NKCC1 at CRH axon terminals in the outer layer of the ME play a pivotal role in CRH release to increase serum corticosterone levels in response to hunger signals.

## Discussion

The classical inhibitory action of GABA in CRH neuronal somata and its significance in regulation of the HPA axis have been studied extensively (Mody and Maguire, 2011; Levy and Tasker, 2012). Previously, we reported a novel excitatory action of GABA at the CRH neuronal axon terminals in the ME (Kakizawa et al., 2016). In addition, we demonstrated that this excitatory action of GABA is not involved in the physiological acute stress response, but rather in steady-state regulation of the HPA axis. Because of the close proximity of the ARC regulating energy balance and food intake (Minor et al., 2009) to the ME with its diverse GABAergic neuronal populations, we hypothesized that they may be involved in activation of the HPA axis by energy homeostasis and feeding responses (Makimura et al., 2003). In the present report, we demonstrate the possible involvement of AgRP-GABAergic neurons within the ARC in CRH release in response to dietary insufficiency.

To demonstrate an association between the HPA axis and energy metabolism, an FR protocol was used. Based on previous reports of FR affecting circulating corticosterone levels (Kenny et al., 2014; Allen et al., 2019) 60% FR for 10 days was performed, which caused increases in serum corticosterone levels (Figure 3C). To ascertain if the enhancement of corticosterone levels was dependent on CRH neuronal activation at the level of somata in the PVN, c-Fos staining was performed. The absence of c-Fos expression in CRH neuronal somata in the PVN indicated CRH neuronal activation was not involved (Figure 3D), suggesting an altogether different mechanism for the observed increase in corticosterone levels.

Diverse GABAergic neurons in the ARC are involved in many critical homeostatic mechanisms, from food intake to fertility (Marshall et al., 2017; Suyama and Yada, 2018). Among them, almost all AgRP neuronal populations co-express GABA and are orexigenic (Horvath et al., 1997; Cowley et al., 2001). Various studies indicate that fasting induces strong activation of AgRP neurons (Takahashi and Cone, 2005; Liu et al., 2012) while FR significantly increases AgRP mRNA (Minor et al., 2009; Rogers et al., 2016). In the present study, 60% FR caused c-Fos activation in AgRP neurons in the ARC (Figures 3E,F), as well as increased circulating corticosterone levels (Figure 3C). However, c-Fos expression was absent in CRH neuronal somata (Figure 3D). Consistent with previous reports (Atasoy et al., 2012; Fan et al., 2021), *in vivo* CNO administration to selectively activate AgRP neurons also induced a clear enhancement of c-Fos expression in AgRP neurons in the ARC (Figures 2A,B) and increased circulating corticosterone levels (Figure 2C), indicating a clear link between AgRP neuronal activation and increased serum corticosterone. In contrast to FR, activation of AgRP neurons by CNO was more robust and CRH neuronal somata were activated, as indicated by intense c-Fos expression in the PVN (Figures 2D,E). Clearly, only a portion of chemogenetically activated AgRP neurons were also activated by FR (compare Figures 2B, upper, 3F; Supplementary Figure 3C).

Thus, our results indicate that increases of serum corticosterone levels by chemogenetic and FR-mediated activation of AgRP neurons were different (compare Figures 2C, 3C; Supplementary Figure 3D). Specifically, we found that FR elevated serum corticosterone levels without affecting CRH neuronal somata in the PVN. Comparison of numbers of activated AgRP neurons and circulating corticosterone levels between chemogenetic activation and FR groups revealed that a large proportion (91.1% ± 2%) of AgRP neurons were activated by CNO administration (Figure 2B, upper), whereas only half (51.3% ± 1.7%) of AgRP neurons were activated by FR (Figure 3F). Similarly, serum corticosterone levels were increased two-fold in CNO-treated group compared with the FR group (Supplementary Figure 3D). Our findings are consistent with the recent study (Fernandes et al., 2022) where they compared both a 24 h and a 36 h fasting protocol. 36 h fasting elevated the mRNA expression of CRH in the PVN and AgRP in the ARC like our CNO administration, whereas 24 h fasting increased AgRP mRNA in ARC but not CRH mRNA in PVN like our FR protocol. The increased corticosterone level is also higher in the 36 h fasting group than the 24 h group, Since the expression of AgRP mRNA in ARC is also higher in the 36 h fasting, the increased corticosterone level is dependent on AgRP neuronal activation. Thus, above difference between CNO and FR might be due to a difference in a population of activated AgRP neurons. Consistent with this, several previous reports using FR protocol found that CRH mRNA expression was not substantially different from the Ad-lib control group in PVN, while AgRP mRNA expression level was increased (de Rijke et al., 2005; Lindblom et al., 2005; Gallardo et al., 2014).

**FIGURE 6:**
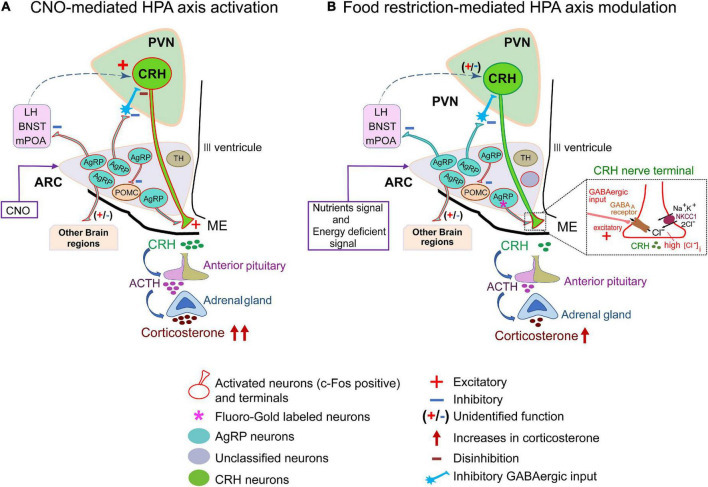
Hypothetical model of hypothalamic-pituitary-adrenal axis activation by food restriction. **(A)** Schematic diagram of CNO-mediated HPA-axis activation. Agouti-related peptide (AgRP) neurons project to diverse brain regions such as the PVN, BNST, LH, mPOA and some other regions. CNO activates almost all subsets of AgRP neurons (91.1% ± 2%; Figure 2B) in ARC to affect their downstream projecting neurons. As a result, about half of corticotropin-releasing hormone (CRH) neurons in the PVN (45.6% ± 1.9%; Figure 2E) were activated and serum corticosterone levels were increased (326.5 ± 21.1 ng/ml; Figure 2C). Dashed arrow line (blue) indicates proposed pathways of CRH neuronal activation. **(B)** Schematic diagram of food-restricted model: a subset of AgRP neurons in arcuate nucleus (ARC) (about 30%, Figure 4C) directly project to the ME. About half (51.3% ± 1.7%) of AgRP neurons are activated by FR (Figure 3F). Among them, about 70% of ME-projecting AgRP neurons were activated by FR (Figure 4E), exhibiting excitation of CRH terminals to increase CRH release and serum corticosterone levels (140.4 ± 18.6 ng/ml; Figure 3C). This excitatory gamma-aminobutyric-acid (GABA) action is promoted by NKCC1 at CRH neuronal terminals (Kakizawa et al., 2016), so that NKCC1 deletion resulted in significant depression of FR-induced corticosterone release (inset). AgRP, agouti-related peptide; BNST, bed nucleus of the stria terminalis; CNO, clozapine N-oxide; CRH, corticotropin-releasing hormone; FR, food restriction; HPA, hypothalamic-pituitary-adrenal axis; LH, lateral hypothalamus; ME, median eminence; mPOA, medial preoptic area; PVN, paraventricular nucleus; TH, tyrosine hydroxylase; POMC, Proopiomelanocortin.

AgRP neuronal axon terminals project from the ARC to various brain regions, such as the PVN, LHA, BNST, raphe nuclei, nucleus accumbens, parabrachial nucleus, periaqueductal gray, paraventricular thalamic nucleus, central nucleus of the amygdala, and brainstem. Therefore, AgRP neurons may be further categorized into differential subpopulations based on their regions of projection (Betley et al., 2013). Indeed, it is quite possible that these projections are associated with different physiological functions and therefore, attributable to differential activation of AgRP neuronal subpopulations (Betley et al., 2013). As previously reported, chemogenetic activation of AgRP neurons may affect multiple downstream brain regions (Steculorum et al., 2016); i.e., CNO administration activates all the AgRP neuronal subpopulations and their downstream targets. CRH neurons in the PVN receive GABAergic input from the peri-PVN, LHA, mPOA, and BNST (Levy and Tasker, 2012). However, a direct synaptic connection between AgRP neuron terminals and CRH neuronal somata in the PVN has not been observed (Garfield et al., 2015). Thus, CRH neuronal somata in PVN could be indirectly activated via inhibition of local inhibitory interneurons by AgRP neurons, hence this disinhibition-dependent activation of CRH neurons may increase circulating corticosterone levels by chemogenetic stimulation (Figure 6A).

In contrast, 60% FR did not activate CRH neuronal somata in the PVN, although serum corticosterone levels were elevated. In addition, FR activated only a subset of AgRP neurons. Thus, it is quite likely that these activated neurons are exclusively involved in energy homeostasis triggered by FR, hence the projections from these neurons might not activate PVN CRH neurons (Figure 6B). Our data also indicate that FR activated ARC AgRP neurons in addition to other local neurons in the ARC. Several types of neurons besides AgRP neurons are present in the ARC, including subpopulations expressing POMC, TH, rat insulin II gene promoter, nitric oxide synthase, and prepronociceptin, which are all GABAergic and related to energy homeostasis. However, FR did not activate TH neurons or POMC neurons (Supplementary Figure 4). In addition, due to the extensive connections of AgRP neurons with other local cells in the ARC (Turi et al., 2003; Cone, 2005; Padilla et al., 2017; Jais et al., 2020; Smith et al., 2020; Varela et al., 2021), we cannot completely rule out the contribution of other neuronal populations in modulating circulating corticosterone levels under FR. Therefore, both the identities and overall function of other local neurons in the ARC interacting with AgRP neurons in response to FR remain to be elucidated.

Corticotropin-releasing hormone neuronal axon terminals exhibit very dense projections to the external layer of the ME (Figure 1B). Furthermore, CRH neuronal axon terminals in the ME express GABA_*A*_ receptors, and GABAergic inputs to ME originate from the ARC (Kakizawa et al., 2016). AgRP neurons are GABAergic and retrogradely labeled by Fluoro-Gold, suggesting that AgRP neurons project to the ME (Figure 1A). Our findings that AgRP neuron terminals project to both the internal and external layers of the ME (Figures 1B,C) are corroborated by the previous report (Bagnol et al., 1999). Our double immuno-electron microscopic analysis further confirmed the presence of GABA vesicles in AgRP axons projecting to the external layer of the ME, whereby they formed symmetric synapses with CRH axon terminals (Figure 1C). We found that 27.72 ± 0.95% of AgRP neurons were labeled by Fluoro-Gold, indicating that a specific subgroup of AgRP neurons projects to the ME (Figure 4C). FR activated approximately half (53.43 ± 3%) of all AgRP neurons examined (Figure 4D), 36.84% ± 2.58% of which were retrogradely labeled by Fluoro-Gold (Figure 4F). These results indicate that this population of AgRP neurons directly projecting to the ME could contribute to modulation of CRH release from CRH neuronal axon terminals in response to FR (Figure 6B). This hypothesis has been supported by the very recent finding that inhibition of AgRP neurons in ARC in fasted animals increases CRH accumulation in the ME, indicating a reduction of release (Fernandes et al., 2022).

Gamma-aminobutyric-acid mediates either inhibition or excitation depending on KCC2 and NKCC1 expression, and functional balance (Rivera et al., 1999; Payne et al., 2003). We previously demonstrated that NKCC1, but not KCC2, was expressed in CRH axon terminals in the ME. In contrast, the somata of CRH neurons were enriched with KCC2 but not NKCC1 (Kakizawa et al., 2016). To evaluate whether AgRP neuronal activation caused GABA-mediated excitation of CRH neuronal terminals in an NKCC1-dependent manner to increase corticosterone levels, conditional knockout of NKCC1 in CRH neurons was performed (Figure 5A). NKCC1 KO^CRH^ mice showed equivalent body weight to WT mice under Ad-lib and 60% FR (Figure 5B). Moreover, the Ad-lib condition produced no difference in circulating serum corticosterone levels between WT and NKCC1 KO^CRH^ mice. Here, NKCC1 knockout was performed in developmental age, presenting a possibility that developmental compensatory mechanism may be involved. The similar serum corticosterone levels in WT littermates and NKCC1 knockout mice in *ad libitum* condition attest to such a possibility. However, the degree by which serum corticosterone levels increased was significantly reduced in NKCC1 KO^CRH^ mice compared with WT mice after FR (Figure 5C). Although AgRP neurons were similarly activated by FR and GABAergic transmission appears to be equivalent in both groups, the loss of NKCC1 in CRH neuron terminals resulted in reduced serum corticosterone secretion in response to FR (Figure 5C). Therefore, the excitatory action of GABA at axon terminals of CRH neurons must be dependent on NKCC1. Considering serum corticosterone levels were not different between WT and NKCC1 KO^CRH^ mice in Ad-lib condition, the GABAergic facilitation of CRH release at axon terminals in ME might be promoted exclusively by FR-induced AgRP neuronal activation in the ARC. But this requires further investigation for mechanistic details.

When the HPA axis functions properly, glucocorticoid deals with stress and has anti-inflammatory actions. However, overproduction can negatively impact metabolic, immune, and central nervous systems, subsequently causing physical and psychiatric problems (Sapolsky et al., 2000; Raison and Miller, 2003). Circulating corticosterone concentrations may be influenced by a variety of factors including the time of day, season, age, sex, reproductive state, exercise, and ultraviolet B radiation (Romero, 2002; Dantzer et al., 2014; Slominski et al., 2018). For instance, a circadian rhythm of baseline glucocorticoid concentrations is found in most species (Romero and Remage-Healey, 2000; Chrousos et al., 2009). Interestingly, evidence suggests that corticosterone secretion is positively related to the diurnal rhythm of AgRP mRNA expression and food intake (Lu et al., 2002). In our study, both chemogenetic activation and FR-mediated activation of AgRP neurons increased corticosterone levels, confirming the involvement of AgRP neurons in corticosterone secretion. This role of AgRP neurons appears to be a critical link between energy demand dependent activation of HPA axis. The resulting disruption of several physiological processes, such as inflammatory response, autonomic function, and neuroendocrine dysregulation, predisposes to metabolic and stress-related disorders. (Harrell et al., 2016).

In the present study, we found that a subpopulation of AgRP neurons activated by FR is responsible for increasing serum corticosterone levels via GABAergic excitation at CRH neuron terminals in the ME (Figure 6B). AgRP neurons are related to feeding and energy homeostasis. Therefore, our novel finding that a specific group of ARC AgRP neurons directly projecting to the CRH axonal terminals in the ME are responsive to fasting signals and involved in reactive secretion of corticosterone via NKCC1-dependent excitatory GABA action sheds light on interactions between the HPA axis and food intake.

## Data availability statement

The original contributions presented in this study are included in the article/Supplementary material, further inquiries can be directed to the corresponding author.

## Ethics statement

All experiments were performed in accordance with guidelines issued by the Hamamatsu University School of Medicine on the ethical use of animals for experimentation, and were approved by the Committee for Animal Care and Use (Approval Nos. 2017056, 2017057, 2018025, and 2020074).

## Author contributions

AF and RY contributed to the conceptualization of the study and wrote the original draft. RY performed the experiments. MW and RY generated the genetically modified mice. AF and MW supervised the study. RY, AF, and AS analyzed the data. AS, MW, MI, TW, AF, and RY contributed to the writing review and editing. All authors contributed to the article and approved the submitted version.
