# Supplementary material for: A subpopulation of agouti-related peptide neurons exciting corticotropin-releasing hormone axon terminals in median eminence led to hypothalamic-pituitary-adrenal axis activation in response to food restriction
Source: Front Mol Neurosci. 2022 Sep 29;15:990803. doi: 10.3389/fnmol.2022.990803 (PMC9557964; doi:10.3389/fnmol.2022.990803)
Supplement: Supplementary file 1 [file Data_Sheet_1.pdf]

Supplementary Material

Supplementary Material

Supplementary Figures

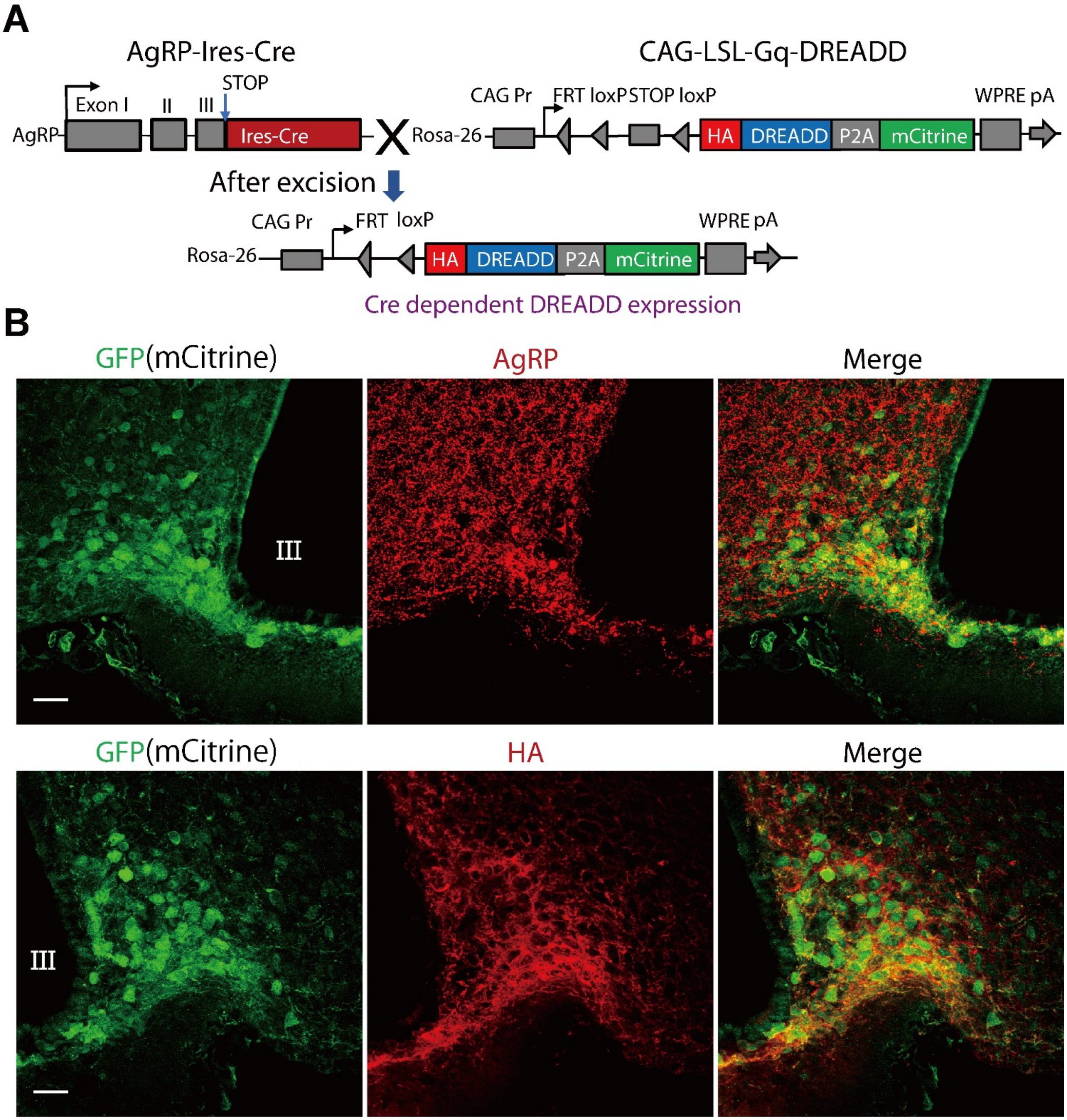

Supplementary Figure 1: Production of mice for selective chemogenetic activation of AgRP neurons; Related to Figure 2. (**A**) Schematic diagram of AgRP-Cre::DREADD transgenic mouse

generation. R26-LSL-hM3Dq-DREADD mice were crossed with AgRP-Ires-Cre driver mice. Note that Cre recombinase-mediated removal of the floxed-STOP cassette results in expression of two proteins: the yellow-green fluorescent protein mCitrine and hemagglutinin (HA) epitope tag. (**B**) Coronal sections of the brain of hypothalamic ARC in AgRP-Cre::DREADD mice stained for mCitrine and HA. GFP antibody was used for mCitrine staining. Upper panel indicate colocalization of mCitrine (GFP) and anti-AgRP immunoreactivity in the ARC. Lower panel indicate confocal images of HA-tagged Gq-DREADD at the membrane of mCitrine positive neurons. Scale bars, 40 µm.

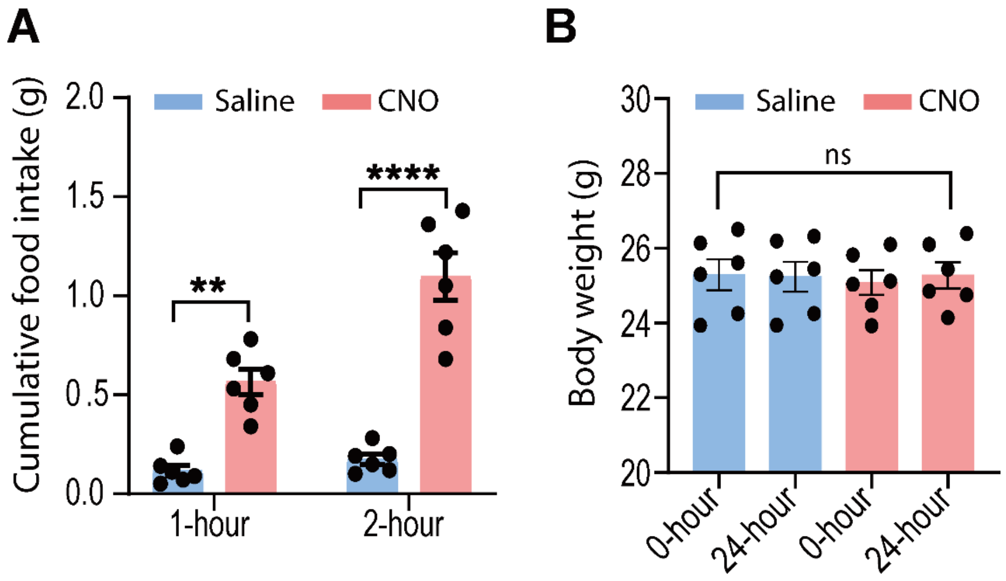

Supplementary Figure 2: Chemogenetic activation of AgRP neurons increased acute food intake; Related to Figure 2. (A) Cumulative food intake after a single injection of CNO or saline in AgRP-Cre::DREADD mice (n = 6 male mice in each group; **P < 0.0013 and ****P < 0.001, two-way ANOVA with Tukey's multiple comparison test). (**B)** Body weight measurement after CNO or saline injection (n = 6 mice in each group). Data were analyzed with two-way ANOVA with Tukey's multiple comparison test.

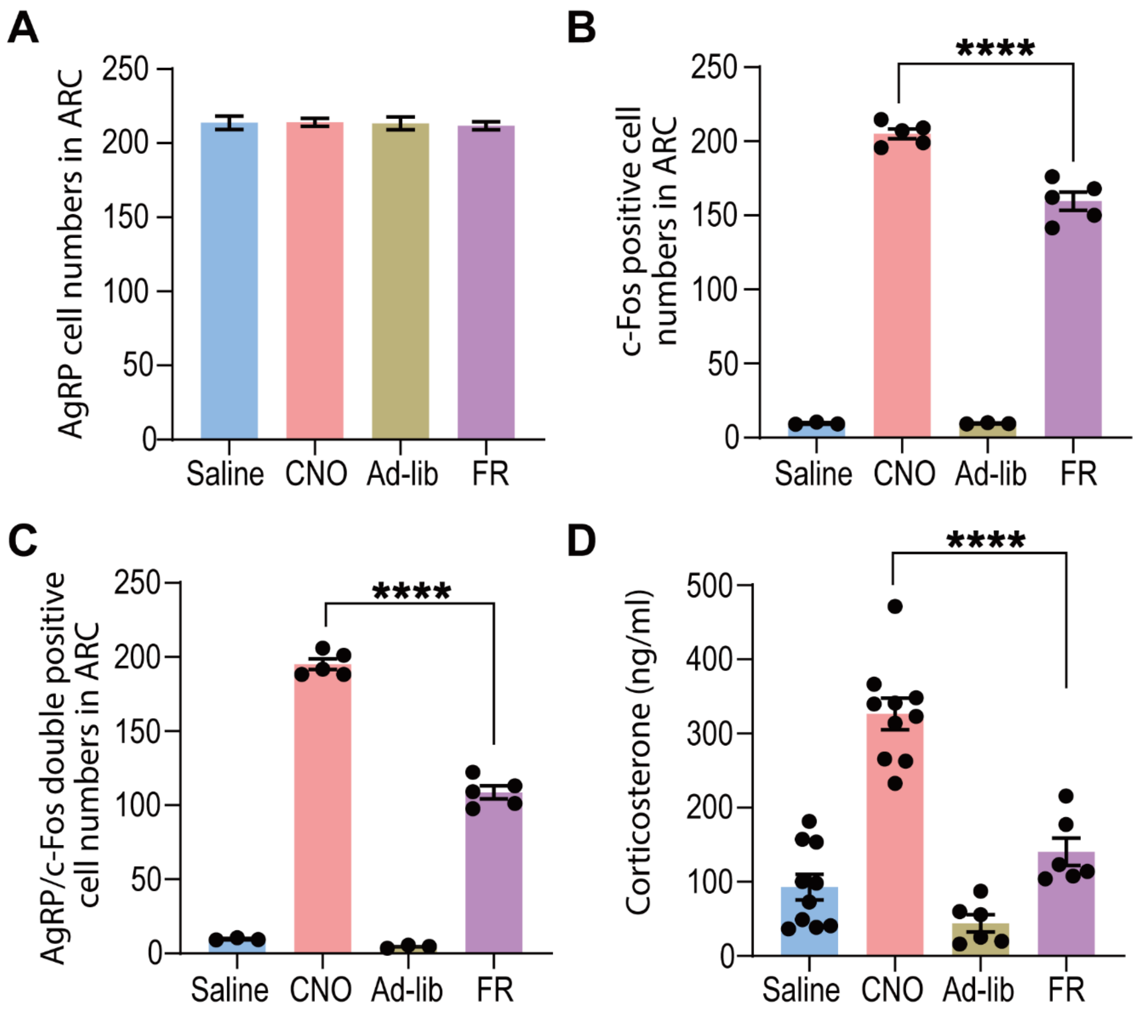

Supplementary Figure 3: Comparisons of numbers of AgRP neurons, c-Fos positive cells, and serum corticosterone levels in saline, CNO, Ad-lib, and food restriction (FR) conditions; Related to Figure 2 and Figure 3. (**A**) Total numbers of AgRP neurons in the ARC in each condition. (**B**) Total numbers of c-Fos-positive cells in the ARC; ****P < 0.0001, two-way ANOVA followed by Tukey's multiple comparison test. (**C**) Numbers of AgRP and c-Fos double-positive cells in the ARC; ****P < 0.0001, two-way ANOVA followed by Tukey's multiple comparison test. (**D**) Comparison of corticosterone levels between CNO and FR groups; ****P < 0.0001, Bonferroni multiple comparison test (D). Error bars represent SEM.

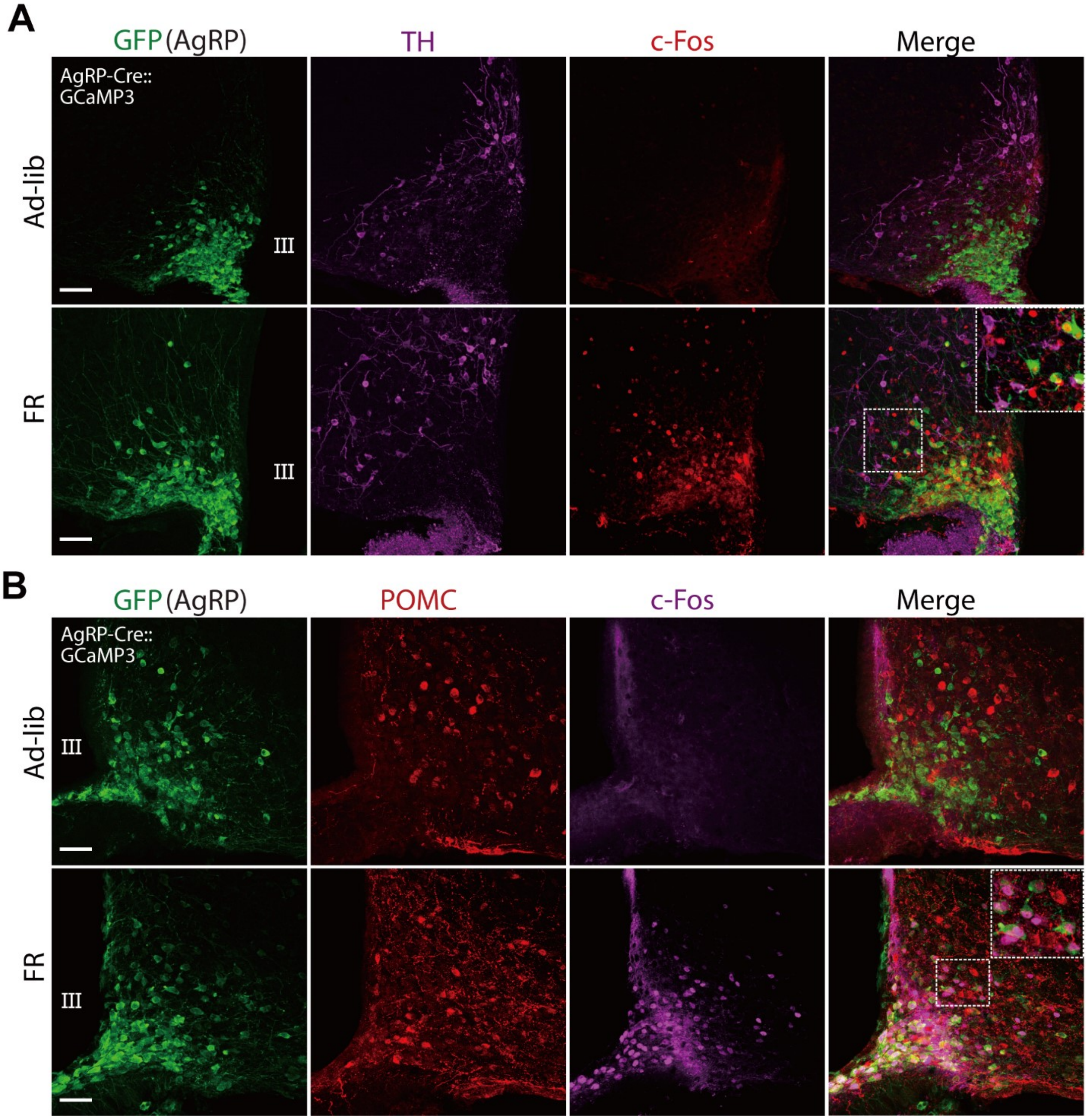

Supplementary Figure 4: Food restriction (FR) did not activate TH or POMC neurons in the ARC; Related to Figure 4. Coronal sections of the ARC are shown. (**A**) Cells were immunostained for AgRP (green), tyrosine hydroxylase (TH, magenta), and c-Fos (red) in Ad-lib (upper panel) and FR (lower panel) conditions. For inset, the dotted boxed area indicates higher magnification image (×4). Scale bars, 40 µm. **(B)** Triple-immunostaining of AgRP (green), POMC (red), and c-Fos (magenta) in Ad-lib (upper panel) and FR (lower panel) conditions. For inset, the dotted boxed area indicates higher magnification image (×4). Scale bars, 40 µm. n = 3 mice in each group. Note that c-Fos-positive cells induced by FR were mostly AgRP neurons.
