# Supplementary figures and images for: A subpopulation of agouti-related peptide neurons exciting corticotropin-releasing hormone axon terminals in median eminence led to hypothalamic-pituitary-adrenal axis activation in response to food restriction
Source: Front Mol Neurosci. 2022 Sep 29;15:990803. doi: 10.3389/fnmol.2022.990803 (PMC9557964; doi:10.3389/fnmol.2022.990803)

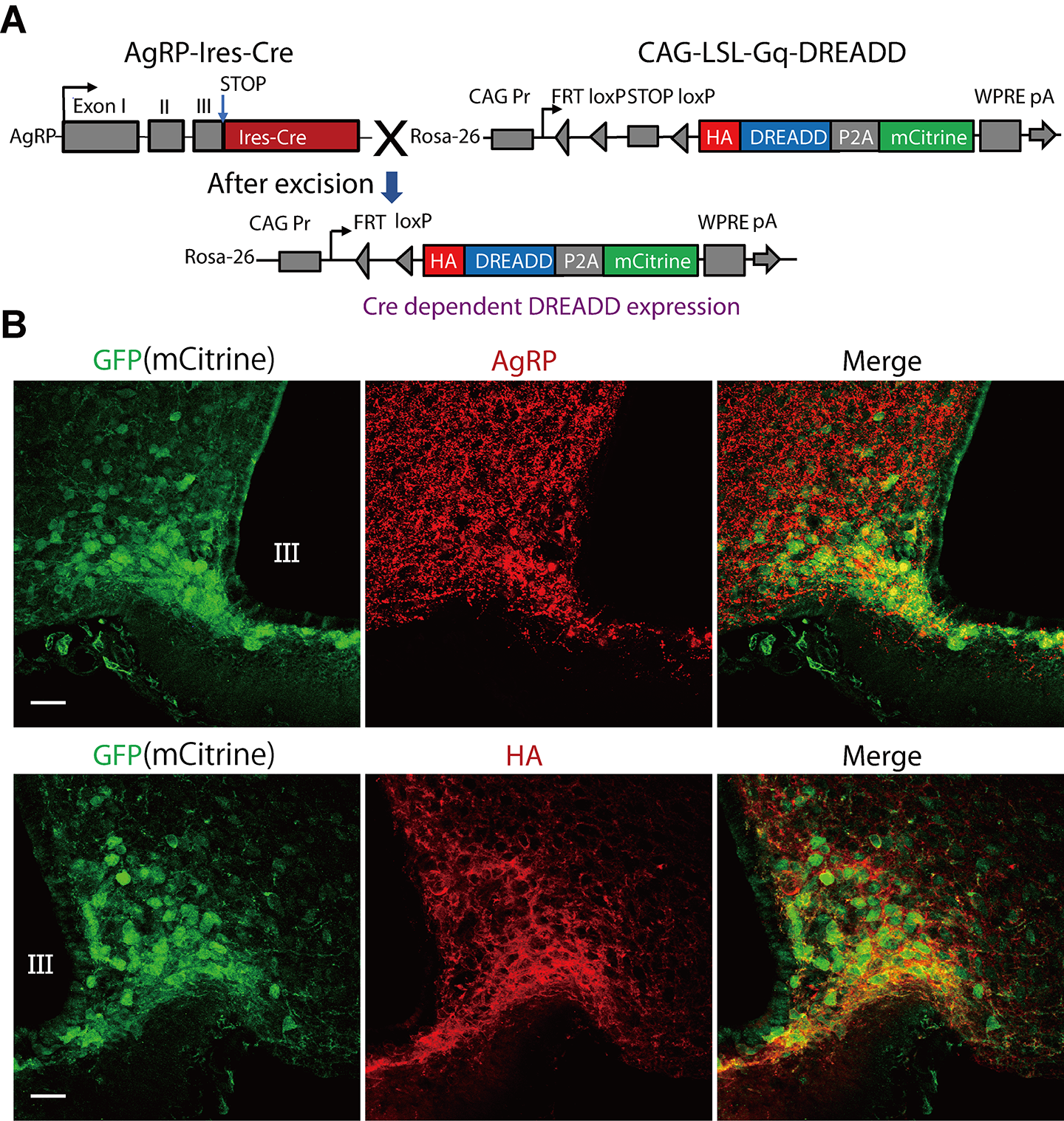

Supplement: Supplementary file 2 [file Image_1.TIFF]

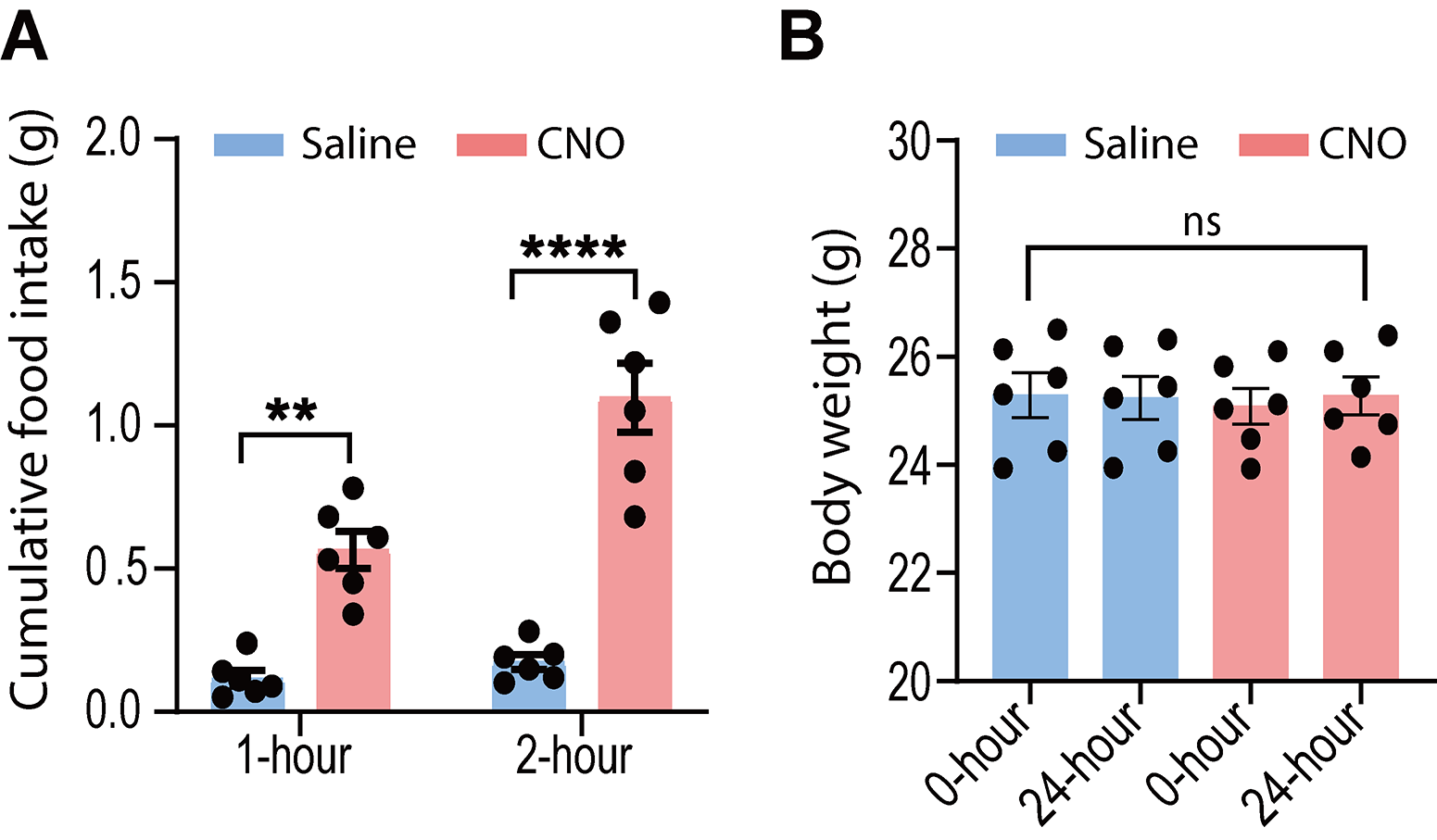

Supplement: Supplementary file 3 [file Image_2.TIF]

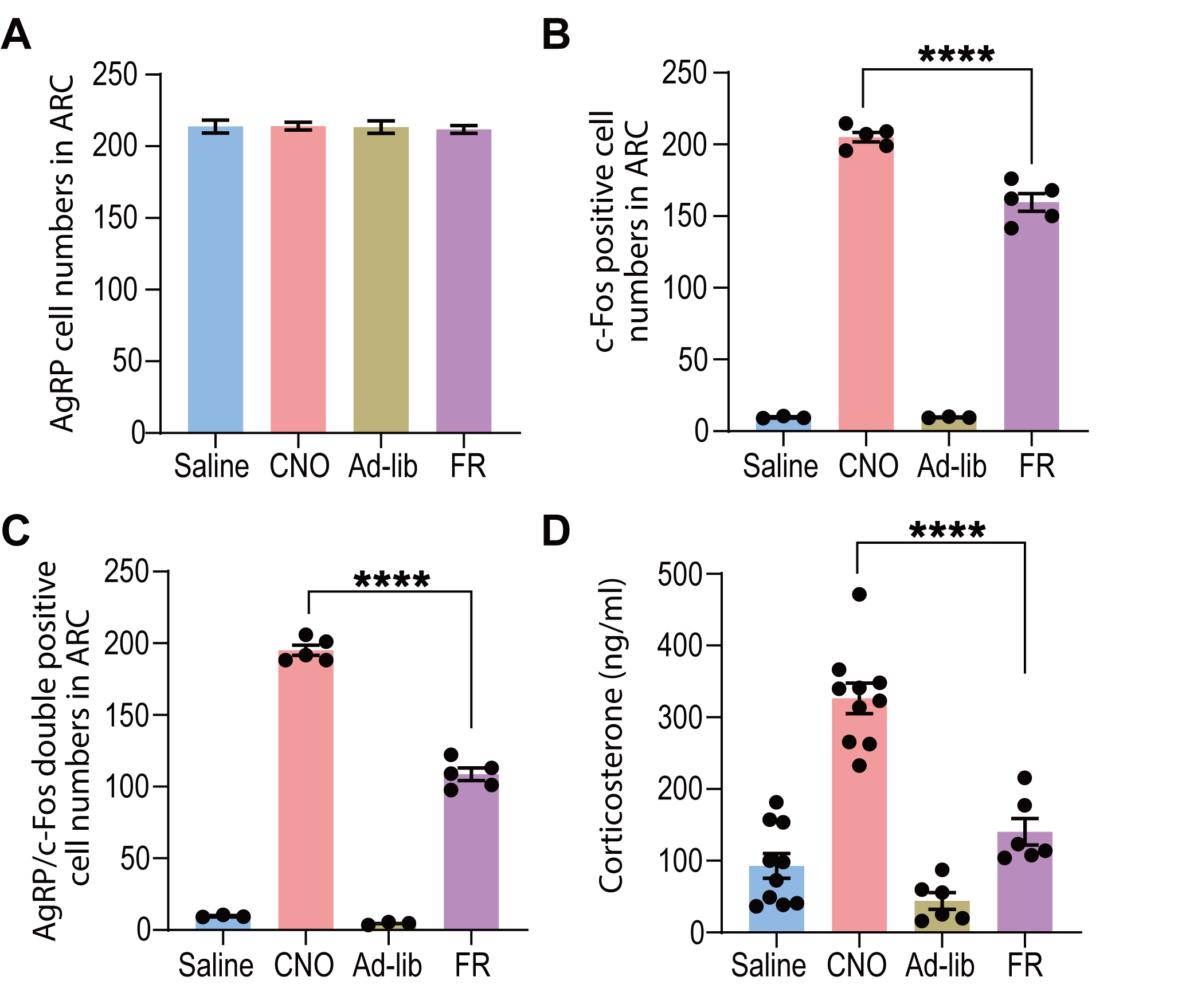

Supplement: Supplementary file 4 [file Image_3.TIF]

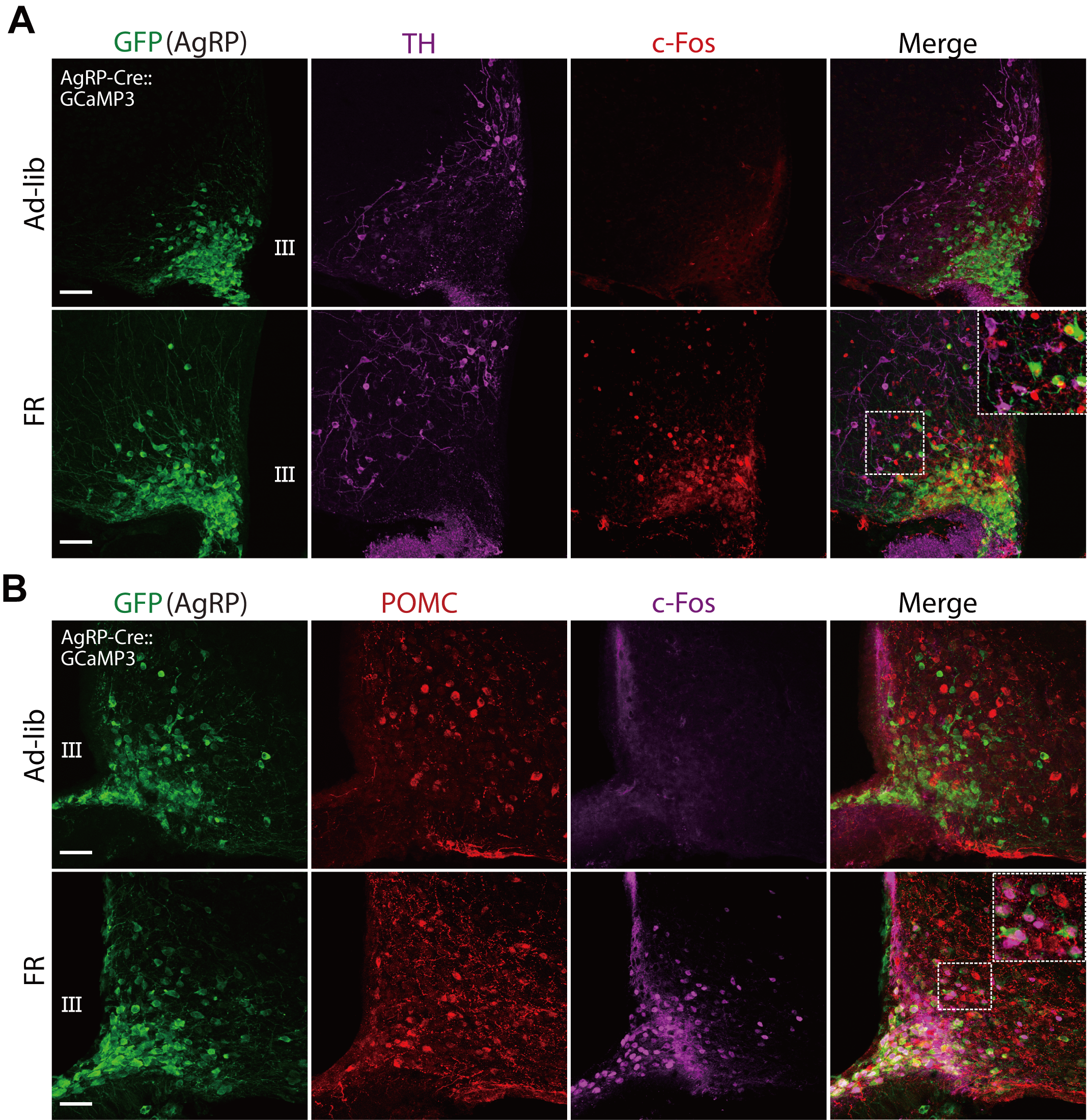

Supplement: Supplementary file 5 [file Image_4.TIF]
