# Supplementary material for: A subpopulation of agouti-related peptide neurons exciting corticotropin-releasing hormone axon terminals in median eminence led to hypothalamic-pituitary-adrenal axis activation in response to food restriction
Source: Front Mol Neurosci. 2022 Sep 29;15:990803. doi: 10.3389/fnmol.2022.990803 (PMC9557964; doi:10.3389/fnmol.2022.990803)
Supplement: Supplementary file 6 [file Table_1.docx]

Supplementary Material

**Supplementary table 1**: Number of AgRP expressing neurons that were stained with c-Fos or Fluoro-Gold or both in food restriction (FR) condition.

| Total mice analysis | Total AgRP^+^ Neurons | AgRP^+^; c-Fos^+^  Neurons | AgRP^+^; Fluro-gold^+^ Neurons | AgRP^+^; c-Fos^+^; Fluro-gold^+^ Neurons |
| --- | --- | --- | --- | --- |
| n=4 | 108.43±3.89 | 57.7±2.5  (53.43±3%) | 30.07±1.58  (27.72±.95%) | 21.20±1.51  (19.49±0.82%) |
