# Supplementary material for: A subpopulation of agouti-related peptide neurons exciting corticotropin-releasing hormone axon terminals in median eminence led to hypothalamic-pituitary-adrenal axis activation in response to food restriction
Source: Front Mol Neurosci. 2022 Sep 29;15:990803. doi: 10.3389/fnmol.2022.990803 (PMC9557964; doi:10.3389/fnmol.2022.990803)
Supplement: Supplementary file 7 [file Table_2.docx]

Supplementary Material

**Supplementary table 2**: Resources table for antibodies, reagents and nucleotides.

| REAGENT or RESOURCES | SOURCE | IDENTIFIER |
| --- | --- | --- |
| Antibodies |  |  |
| Guinea pig anti-CRH | Peninsula Laboratories | T-5007, RRID: AB_518256 |
| Mouse anti-c-Fos | Abcam | ab208942, RRID: AB_2747772 |
| Chicken anti-GFP | Abcam | ab13970, RRID: AB_300798 |
| Goat anti-AgRP | R&D system | AF634, RRID: AB_2273824 |
| Rabbit anti-TH | Millipore | Ab152, RRID: AB_390204 |
| Mouse anti-HA | Bio legend | 901501, RRID: AB_2565006) |
| Rabbit anti-c-Fos | Sigma-Aldrich | F7799, RRID: AB_259739) |
| Mouse anti-NKCC1 | DSHB | T4, RRID: AB_528406 |
| Rabbit anti-POMC | Phoenix Pharmaceuticals | H-029-30, RRID: AB_2307442) |
| Rabbit anti-Fluoro-Gold | Merck | AB 153-I, RRID: AB_2632408 |
| Chemical | | |
| Clozapine-N-oxide (CNO) | Sigma-Aldrich | C0832 |
| Fluoro-Gold | Flurocrome |  |
| Critical commercial assay |  | |
| CORT RIA kit | Institute of Isotopes | RK-548 |
| Experimental models: Organisms/strains | |  |
| Mouse: AgRP-IRES-Cre+/- | Jackson Laboratory | JAX: 012899 |
| Mouse: CRH-IRES-Cre+/+ | Jackson Laboratory | JAX: 012704 |
| Mouse: GCaMP3+/+ Ai38 | Jackson Laboratory | JAX: 014538 |
| Mouse: Gq-DREADD+/- | Jackson Laboratory | JAX: 026220 |
| Mouse: NKCC1^flox/flox^ | Dr. Christian A. Hübner | N/A |
| Mouse: C57BL/6J (wild-type) | Japan SLC Inc. | N/A |
| Software and algorithms | | |
| GraphPad Prism 8 | GraphPad | https://www.graphpad.com/ |
| ImageJ | NIH ImageJ | https://imagej.nih.gov/ij/ |
| Confocal microscopy | (Olympus FV1000-D or Leica TCS SP8) | N/A |
| Oligonucleotides | | |
|  |  |  |
| 5'→3' AgRP-C-F: GCTTCTTCAATGCCTTTTGC | RIKAKEN | N/A |
| 5'→3' AgRP-WT-R: GTGTGTGGTTCCAGCATGAC | RIKAKEN | N/A |
| 5'→3' AgRP-MT-R: AGGAACTGCTTCCTTCACGA | RIKAKEN | N/A |
| 5'→3' CRH-C-R: CTTACACATTTCGTCCTAGCC | RIKAKEN | N/A |
| 5'→3' CRH-WT-F: CACGACCAGGCTGCGGCTAAC | RIKAKEN | N/A |
| 5'→3' CRH-MT-F: CAATGTATCTTATCATGTCTGGATCC | RIKAKEN | N/A |
| 5′→3' DREADD-F: ATGTCTGGATCCCCATCAAG | RIKAKEN | N/A |
| 5'→3' DREADD-R: GATGTTGCCGATGATGGTCAC | RIKAKEN | N/A |
| 5'→3' GCaMP3-MT-F: TGGGGATGGTCAGGTAAACT | RIKAKEN | N/A |
| 5'→3' GCaMP3-WT-F: GAGTTCTCTGCTGCCTCCTG | RIKAKEN | N/A |
| 5'→3' GCaMP3-MT-R: CCACATAGCGTAAAAGGAGCA | RIKAKEN | N/A |
| 5'→3' GCaMP3-WT-R: TAAGCCTGCCCAGAAGACTC | RIKAKEN | N/A |
| 5'→3' NKCC1-WT: GCAATTAAGTTTGGAGGTTCCTT | RIKAKEN | N/A |
| 5'→3' NKCC1-F: TGGTGTGAAGGAACAGTTGG | RIKAKEN | N/A |
| 5'→3' NKCC1-R: CCAACAGTATGCAGACTCTC | RIKAKEN | N/A |
